# The USADA’s supposed orthodox approach to anti-doping: a power strategy and a threat to trust

**DOI:** 10.3389/fspor.2025.1619707

**Published:** 2025-07-16

**Authors:** Fabien Ohl

**Affiliations:** Faculty of Social and Political Sciences, REDs (Center of Research and Expertise in Anti-Doping Sciences), Sport Sciences Institute (ISSUL), University of Lausanne, Lausanne, Switzerland

**Keywords:** doxa, field, sociology, power, anti-doping, trust, sport, WADA

## Abstract

This article analyses the roots of the significant tensions between USADA and WADA over the revelations that 23 Chinese swimmers were found to have a banned substance in 2021. USADA described the revelation as a scandal and criticised WADA for a lax policy that failed to fulfil its role. USADA's position, which can be described as a way of overplaying orthodoxy, is seen as an attempt to control the "doxa" of sport and thus gain power within the Olympic movement. This vehement demand for a change in WADA policy can be seen as one of the expressions of the imperialist stance of the United States, which favours power relations over deliberation and multilateralism and risks undermining confidence in anti-doping.

## Introduction

Controversy over the issue of doping is a recurring theme. The identification of state-sponsored doping organised by Russia in Sochi in 2014 has already been the subject of controversy, with the US Anti-Doping agency (USADA) previously calling into question the work of the World Anti-Doping Agency (WADA). In 2024, when journalists revealed the 2021 findings of a banned substance (trimetazidine TMZ) in 23 Chinese swimmers, had not been made public by WADA, USADA accused WADA of not having fulfilled its mission. USADA argued that WADA's integrity and authority had been undermined, and that trust had been eroded. WADA denied these allegations. Tensions between the two organizations ran high, as evidenced by defamation lawsuits and the suspension of US's contribution to WADA's funding. This article takes a step back to analyse these differences and place them in the context of the historical dynamics and geopolitical issues of anti-doping.

## Doping as a social and legal norm

To understand these controversies, it is important to remember that doping is not a universal norm; it is rooted in history and was gradually forged over the course of the twentieth century. The use of performance-enhancing drugs was not a prominent issue at the beginning of the 20th century. It was not until the 1960s that the IOC and various governments developed the first definitions and legal rules for doping. The use of doping and its regulation subsequently evolved under the influence of geopolitical issues, particularly the Cold War (1). Anti-doping regulations were gradually harmonized within the Olympic movement with the creation of WADA in 1999.

## Doping at the heart of the Olympic movement's doxa

From a sociological perspective, anti-doping can be identified as a central component of the doxa of sport, in the sense of P. Bourdieu (2), i.e., a “set of fundamental beliefs” whose acceptance is implied by belonging to the same social space. Those involved in sport share the belief that sporting performance (rankings, medals, etc.) gives great value to individuals and nations and constitutes the main capital produced by sport. Doping is a threat to one of the foundations of this doxa, namely that performance is a way of assessing the merits and qualities of athletes.

As a result, doping is seen as a heterodox practice, and the central role of anti-doping is to protect the value placed on performance. But the interpretation of the norm varies from country to country and from sport to sport. For example, American professional leagues have not signed the WADA World Anti-Doping Code, and their rules and sanctions are less stringent.

As in other areas, the control of doxa is at the centre of power stakes (3), and players compete for positions of power to direct WADA's operations. For example, the exclusion of the Russians after the doping scandals at the 2014 Sochi Olympics (4, 5) was the subject of contrasting reactions: those close to Russia considered the exclusion unfounded, while the Americans, with USADA at the forefront, thought that WADA and the IOC had been complacent about Russian state-sponsored doping (6).

## Anti-doping as a confidence-building measure

The fight against doping is not only a technical tool to punish cheats, but also a “confidence-building mechanism” (7), one of the aims of which is to preserve the value of competitions. As a result, anti-doping organizations must demonstrate their credibility and, as in science (8), their communication must be performative, it must inspire confidence by showing convincing results, innovation, unwavering commitment, evidence, and so on.

This trust, which lies at the heart of anti-doping, has been undermined on several occasions. For example, the promise that anti-doping science would lead to clean sport has not been kept. The promises have been excessive and violently contradicted by the facts, notably by the Festina scandal of 1998, which revealed almost systematic doping in professional cycling, and the Russian state-organized doping around the Sochi Olympics in 2014. We have described this orthodoxy as toxic because the untenable promise of doping-free sport is a burden that has fuelled mistrust and discredited anti-doping (6).

The corruption of L. Diack, President of the International Association of Athletics Federation, who accepted bribes to cover up cases of doping, remind us that the determination of certain members involved in the Olympic movement to fight against doping was by no means self-evident. But the global regulation by WADA –in collaboration with some of the national anti-doping agencies, the AIU (Athletic Integrity Unit) (9), the ITA (International Testing Agency), etc.– testifies to the quality of the systems, resources and skills that, without claiming to be perfect, represent a break with the past.

## Heterodoxy and orthodoxy as strategies for gaining power

The lack of trust can have many causes, but the struggles for power within sport play a crucial role. WADA and the Olympic movement, which occupy a central position of power, control and defend the existing doxa, for example by protecting or changing the standards that guide the way performance is produced.

As observed by Bourdieu [(2), p. 102] “All those who are involved in the fields share a tacit adherence to the same doxa”, yet they also compete for control of the doxa by challenging the established order, presenting themselves as defenders of an idealised orthodoxy or venerating the past. As Bourdieu [(10): 417], observed, this idealised vision of a lost world and lost values is often used to contest the established order and gain power. This is, for example, what Tygart is doing by idealising WADA's past, telling that “WADA had a lot of success under Jacques Rogge” [the former IOC president Jacques Rogge (11) to criticize the current situation.^1^

This struggle for power is also expressed in USADA's criticism of WADA's lack of orthodoxy, which allows the case against the Chinese swimmers to be built up as a “scandal”. The roles and decisions of WADA, World Aquatics and CHINADA (China's anti-doping agency) are certainly open to criticism. But it is also understandable that both World Aquatics and WADA should have been convinced by the scientific experts, for example the opinion of Jordi Segura –a recipient of USADA's “Award for Excellence in Anti-Doping Science” at USADA's 2022 Annual Scientific Symposium– who concluded that no hypothesis other than contamination was more likely^2^. Consequently, an appeal of CHINADA's decision to the Court of Arbitration for Sport (CAS) may have strengthened the investigation or provided reassurance, but it was by no means a foregone conclusion. But as in other forms of justice, decisions are linked to a body of evidence, the relationship between the resources invested, the likelihood that an investigation will be able to prove guilt, the risk that an appeal will not succeed, and so on.

Criticism and controversy are necessary for the proper democratic functioning of institutions. But the violence of USADA's accusations (of compromise, complacency, concealment of facts, etc.) and the way USADA has constructed this affair as a scandal comparable to that of Sochi are highly questionable. In Sochi there was a clear motive, because V. Putin and the Russian state wanted to shine at their Olympic Games. What is the motive behind WADA's alleged complacency towards China? There has been talk of Chinese funding exceeding its mandatory contribution^3^, but this point is irrelevant; it is quite common for states to voluntarily and transparently make additional contributions to match the funds allocated by the sports movement for anti-doping.

There's also another inconsistency. If the Chinese had organized doping, they would not have tested these athletes, or they would have verified that the athletes would not be declared positive before conducting official tests. In non-democratic regimes with strong political powers, the possibility of avoiding doping cases is higher when testing is done by their own agency in a national competition than in other countries. The assumption that China's strategy is to report cases to show that the fight against doping is effective and then try to neutralize them is not convincing. To show that you are fighting doping, it is easier to find cases among second-tier athletes.

## The power to challenge power

Other avenues must be explored to explain why this case is being treated as a “scandal” by USADA, because there are no convincing reasons or explanations for WADA's complacency.

The most realistic hypothesis is that of a power struggle. Due to its resources, influence, and power in the field of sports and anti-doping, the USADA has the power to create a “scandal”. USADA's power is primarily symbolic. T. Tygart, USADA's chief executive officer and chief spokesman for its critics, has wielded considerable influence since exposing Lance Armstrong's doping by arriving at the right moment (2012) and capitalising on early witnesses, cyclists and critical journalists, as well as an FBI investigation, to achieve one of the most significant results in the fight against doping, earning him considerable symbolic capital and political clout. While these power struggles may be linked to interpersonal rivalries and Tygart's personality, these factors alone cannot explain the scale of the clashes. On one hand Tygart enjoys political support from R. Gupta, Former Director of the Office of National Drug Control Policy, as can be seen in his testimony before the US Senate.^4^ He also benefits from the political support offered by world geopolitics, particularly the stark contrast between the US and China.^5^ On the other hand, Tygart's statements are also powerful because he can rely on the dominance of the United States and its sponsors in the sports economy. These arguments concerning the United States' dominant economic influence within the Olympic movement and accusation against China were clearly expressed during a Senate hearing on 17 June 2025, where more power of the US within WADA was demanded.^6^ In addition, USADA can also count on a network of allied players. In particular, Global Athlete (GA) –an organization, funded by the Fairsport Foundation, that gives a voice to athletes without a mandate to represent them, echoes USADA's position. The current tug-of-war follows on from the positions taken by Global Athlete and FairSport –the same international network previously mobilised in support of the unsuccessful bid for the WADA presidency (12).

In summary, USADA and T. Tygart have amassed significant power that allows them to easily spread the “scandal” narrative.

## The contradictions of the US position

The first breach of the World Anti-Doping Agency's (WADA) global anti-doping regulations occurred with the passing of the *Rodchenkov Act*, which granted the United States extraterritoriality in anti-doping matters in 2020, on the grounds that WADA was not doing enough in this area.

This initiative, taken in the wake of the Russian athletes' doping scandal, is a breach in the harmonisation of anti-doping rules.^7^ It gives the United States the right to sanction anti-doping offences in other countries, thereby bypassing the process of harmonising rules. Similar action by other countries would create uncertainty for athletes and coaches by increasing the risk of doping cases being exploited for political gain.

The U.S. government, citing WADA's perceived lack of orthodoxy, has since refused to contribute to WADA's funding, thereby undermining the anti-doping economy. The logic of trying to improve anti-doping by taking resources away from anti-doping seems questionable. Tygart, who has held considerable power within the USADA for almost 20 years, reiterates that WADA lacks transparency.^8^ However, the comparison with WADA is not favourable. For example, the minutes of all WADA governing body meetings are publicly available, but those of the USADA are not accessible on its website. This means that he is demanding greater transparency in the governance of WADA, even though the organisation he heads is much more opaque. For example, although the “Nominating and Governance Committee” appears to hold central authority within the organisation, its composition and operating rules remain unknown.^9^

Despite this opaque governance, USADA uses the argument of strengthening orthodoxy and “good governance” to fuel a power struggle that appears to be aimed at gaining influence in international sport. For example, in 2020, in the wake of the Russian state doping scandal, USADA already demanded more power within WADA and threatened to withhold its financial contribution^10^. More recently, the U.S. Congress introduced a bill, the “Restoring Confidence in the World Anti-Doping Agency Act of 2024,” which explicitly calls for “fair representation of the United States” in WADA's governance, while North Americans, like Europeans, have been so far over-represented within the WADA's main committees and governing bodies (13).^11^ There is also an almost messianic view of the role of the United States, a kind of struggle between good and evil that justifies the imposition of the idea that WADA is complicit and that it turns a blind eye to countries like Russia and China.

But the paradox is that the United States also has heterodox positions in terms of anti-doping, since the private American leagues in football, baseball, hockey, basketball, the UFC in MMA, etc. have less strict regulations compared to WADA's rules, and support for *Enhanced Games,*^12^ which authorise doping, is strong. It would be fair to distinguish between the activities of the USADA and those of other parties, as well as to differentiate between the positions of the United States Olympic Committee (USOPC), which was initially aligned with the USADA before adopting a more nuanced position in this power struggle.^13^ However, the political synergy between USADA, the US Congress and Senate cannot be ignored. Although these links need to be clarified, it can be observed that USADA regularly relies on the latter in its power struggle.

Inconsistently, although it claims to want to combat doping internationally through the *Rodchenkov Act* —signed by President Trump— the commitment remains selective, largely excluding the US national level, and is primarily aimed at protecting US interests. Furthermore, the *Enhanced Games* are supported by individuals close to the US president —such as P. Thiel (He is a billionaire entrepreneur, libertarian political activist and key supporter of Trump) and D. Trump Jr. (his son), who are among its main promoters— and its website displays its affinity with Donald Trump's values by featuring a large portrait of him accompanied by the text: “The impossible is what we do best”^14^. Despite the claim to be “separate and independent from the Olympics”, the organisers of the *Enhanced Games* are on the same sport events market and aim to rival the Olympic Games while exposing its alleged hypocrisy on the issue^15^ and echoing the rhetoric of the USADA in its condemnation of WADA^16^.

## Policy proposals that undermine confidence

The scandal caused by the revelations of widespread doping in cycling led to the creation of WADA to restore confidence. The creation of WADA had a performative dimension, with governments and organizations trying to convince people that they were taking the problem seriously. There was also an operational dimension, characterized by the desire to clarify and harmonize the legal framework, improve the collection of evidence, etc. Engaging in a power struggle does not change the mechanisms, the World Code, the various standards, the day-to-day work of those involved in anti-doping, etc., while the withdrawal of funding could even jeopardize these systems. Crucially, it changes the performative aspects of anti-doping. USADA's decision to bring the controversy to the forefront through repeated attacks aimed at a very wide audience, including athletes, governments, the media, etc., is very likely to affect confidence in anti-doping.

As a result, and as we have observed, anti-doping professionals feel negatively affected by the contrast between their determined commitment to doing their job well, their contribution over the years to improving anti-doping daily, and the negative image of anti-doping created by controversies set against a backdrop of geopolitics.

Of course, trust is complex, both essential and difficult to identify. And it must remain limited, because excessive trust could encourage abuse. This is indeed what we see with athletes, whose relationship of trust with anti-doping is ambivalent, with both support and a degree of mistrust (14). But a lack of trust can also be a problem, as it fuels suspicion of competitors, and can remove a brake on athletes' reluctance to dope by facilitating moral disengagement (15). As a result, spreading the idea that the anti-doping system does not work risks becoming a self-fulfilling prophecy.

## Conclusion

Because of its multiple dimensions: political, biochemical, social, economic, symbolic, legal, etc., and because of the need to balance the ability to sanction doping athletes with respect for athletes' rights, anti-doping is highly complex. And yet, because of the many issues at stake and the emotional dimensions of sport, reductive stances, simplistic accusations, and unsubstantiated suspicions are more common than attention to complexity.

There is no doubt that criticism of anti-doping efforts is necessary, and there has been much criticism based on research. For example, WADA has been criticised for its rationale for anti-doping policy and strategy, which is mainly based on biological factors (16); the multitude of environmental demands and constraints induces permanent stress due to the diversity and contradictions of stakeholder demands, thereby impeding decision-making (17); and its effectiveness has also been questioned (13). Our own research activities have also contributed to this by providing an independent scientific perspective [e.g., (6, 18)]. However, like journalists, researchers may be inclined to adopt a critical stance towards powerful institutions. Nevertheless, we must also be wary of such simplistic views, as they can fuel conspiracy theories. Without being naive, we must criticise the critics and identify why no critical analysis is being produced. For instance, despite some research (19–21), very little research has been conducted into other anti-doping organisations, particularly national agencies. Yet this is essential to understanding how anti-doping works. Criticism is all the more important because it helps to avoid overconfidence, which is not conducive to questioning standards, their regulation, and their evolution.

But instead of contributing to constructive criticism, USADA's violent attacks threaten confidence in anti-doping. By emphasising its orthodox stance to such an extent, USADA is following in the footsteps of American policies that prioritise force over multilateralism and deliberation, as evidenced by the United States' numerous official withdrawals from international organizations such as the WHO, the IPCC, UNRWA, etc.^17^ In line with these changes, USADA seems attempting to exploit the current culture of mistrust and suspicion towards institutions (22).

However, as Quéré reminds us, trust is not a weakness, but rather a form of intelligence. We should therefore dare to trust more -which would not be out of place given the changes in anti-doping over the past 25 years- while remaining highly critical, disbelieving and vigilant.

## Actionable recommendations

Based on this reflection, we make three recommendations to inform the discussion on improving anti-doping measures and maintaining confidence in the system:

1. When faced with sensitive cases, WADA could involve representative of other trustworthy anti-doping organisations in the decision-making process, subject to conditions relating to conflicts of interest, integrity and confidentiality. While this more participatory model presents risks such as complexity, bureaucracy and power struggles, it can also help to develop a more collective culture, strengthen the legitimacy of decisions and build stakeholder confidence.
2. Although the fight against doping now has more realistic objectives (rather than trying to eradicate doping, the focus is on protecting “clean” athletes), expecting perfect decisions or perfectly comparable sanctions is incompatible with true justice, which must consider the unique circumstances of each case. To prevent an escalation of orthodoxy that undermines confidence in the fight against doping, Olympic movement stakeholders must change their idealistic view of the fight against doping, recognise its limitations and uncertainties, and accept its complexity. This cultural change must be implemented in sports organisations to think about anti-doping beyond the binary opposition between cheats and clean athletes.
3. Critical research on anti-doping organisations is important for improving their governance. However, such research tends to focus primarily on WADA, even though other anti-doping organisations also play a crucial role in the fight against doping. The lack of critical research in this area is a significant shortcoming.

## Footnotes

^1^He cannot ignore the fact that Rogge – who was a close friend of Hein Verbruggen, the former president of the UCI who was a staunch supporter of Lance Armstrong– never publicly criticised the UCI or Verbruggen for their complacency, even after the USADA report officially accused them in 2012. See the 2015 report by the Cycling Independent Reform Commission (CIRC, p. 93), https://velo-cdn.outsideonline.com/wp-content/uploads/2015/03/CIRC-Report-2015.pdf (Accessed on 2025.06.09)

^2^See https://www.wada-ama.org/sites/default/files/2024-09/202408_final_cottier_report_english_translation.pdf p.10

^3^See US Senate Hearings (2025.06.17) WADA Shame: Swimming in Denial Over Chinese Doping https://www.commerce.senate.gov/services/files/79B1679D-1F20-4C3D-B25C-65D1C79EC6FE p.4

^4^https://www.commerce.senate.gov/services/files/91791234-D0DE-4EEA-A768-53DCDB64E360 Accessed 2025.06.17

^5^For example, John Moolenaar, who supported this fight in the US Congress, is a congressman who "also serves as Chairman of the House Select Committee on the Strategic Competition Between the United States and the Chinese Communist Party" see https://moolenaar.house.gov/committees-and-caucuses

^6^See US Senate Hearings 17 June 2025 WADA Shame: Swimming in Denial Over Chinese Doping https://www.commerce.senate.gov/2025/5/wada-shame-swimming-in-denial-over-chinese-doping_2_2_2

^7^ https://www.insidethegames.biz/articles/1095977/ulrich-haas-blog-rodchenkov-act

^8^This is a recurring theme, among many examples the US Senate Hearings https://www.commerce.senate.gov/services/files/79B1679D-1F20-4C3D-B25C-65D1C79EC6FE

^9^See https://www.usada.org/wp-content/uploads/USADA_Bylaws-2023.pdf Accessed 2025.04.09

^10^See Minutes of the WADA Foundation Board Meeting, 2020.11.12, pp.21-25 consulted 12.02.2024 https://www.wada-ama.org/sites/default/files/resources/files/minutes_foundationboardnovember2020_final.pdf

^11^G:\M\18\MOOLEN\MOOLEN_099.XML, 118TH CONGRESS of the United States, 2D SESSION, July 29, 2024, from John Moolenaar and the US Senate continued in the same vein on 17 June 2025 see https://www.commerce.senate.gov/2025/5/wada-shame-swimming-in-denial-over-chinese-doping_2_2_2. However, a country which neither pays its financial contributions nor refrains from launching scathing attacks against the organisation cannot expect to play a central role in its governance.

^12^These Games, which are scheduled to take place in Las Vegas for the first time in 2026, allow the use of science and technology to enhance performance. This means there are no restrictions on doping see https://www.enhanced.com

^13^See https://www.usopc.org/news/2024/april/22/statement-regarding-the-chinese-swimming-investigation and https://www.usopc.org/news/2025/april/17/audio-april-2025-usopc-leadership-press-briefing.

^14^https://www.enhanced.com/series-b Accessed 2025.04.10

^15^https://www.foxnews.com/sports/enhanced-games-founder-says-steroid-approved-event-future-sports-calls-out-hypocrisy-olympics Accessed 2025.04.10

^16^https://www.enhanced.com/newsroom/enhanced-games-president-call-for-reform-amidst-wada-s-crisis-of-trust Accessed 2025.04.14

^17^Despite numerous appeals –for example https://www.theguardian.com/sport/2025/jan/15/uk-and-eu-urged-to-follow-us-and-block-funding-for-world-anti-doping-agency– to our knowledge no other iNADO (International Group of National Anti-Doping Organisations) agency has aligned itself with the American position.
